# Adsorption of Phosphate by Two-Step Synthesis of Ceramsite from Electrolytic Manganese Residue/Dredged Sludge

**DOI:** 10.3390/ma17040939

**Published:** 2024-02-17

**Authors:** Hao Cheng, Wei Shi, Song Liu, Yong Wang, Jia Song, Yu Long, Yuan Xiang, Yongjie Xue

**Affiliations:** 1 College of Material and Chemical Engineering, Tongren University, Tongren 554300, China; smalone.1@163.com (H.C.);; 2 State Key Laboratory of Silicate Materials for Architectures, Wuhan University of Technology, Wuhan 430070, China

**Keywords:** electrolytic manganese residue, dredged sludge, ceramsite, low-temperature preparation, phosphorus adsorption, adsorption mechanism

## Abstract

Carrying out research on the management of electrolytic manganese residue (EMR) is necessary to maintain the environment and human health. The dredged sludge (DS) and water hyacinth (WH) generated from dredging projects are potential environmental threats, and therefore suitable methods need to be found for their treatment. In this study, ceramsite was prepared by a two-step low-temperature firing method using DS and EMR as raw materials, WH as a pore-forming additive, and aluminate cement as a binder for the adsorption of phosphorus from wastewater. The optimal ratio and process parameters of the ceramsite were determined by mechanical and adsorption properties. The static adsorption experiments were conducted to study the effect of ceramsite dosage and solution pH on the removal of phosphorus. At the same time, dynamic adsorption experiments were designed to consider the influence of flow rate on its actual absorption effect, to explore the actual effect of ceramsite in wastewater treatment, and to derive a dynamic adsorption model that can provide technical support and theoretical guidance for environmental management.

## 1. Introduction

Phosphorus is a crucial resource with widespread applications in both agriculture and industry. Nevertheless, the presence of substantial phosphate concentrations in wastewater remains a primary contributor to eutrophication in aquatic ecosystems, a phenomenon associated with enduring environmental complications in lakes and coastal waters [1]. The main treatment methods commonly used are chemical precipitation, adsorption, ion exchange, and electrolysis. Among these methods, the adsorption method is the most widely used. The key to the adsorption method is the design and preparation of efficient adsorbents. Ceramsite has outstanding advantages, such as a high specific surface area and porosity, so it can be used as adsorbents for phosphorus-containing wastewater [2]. In recent years, the development of ceramsite through the co-disposal of multiple solid wastes has gained prominence. For instance, Yin et al. [3] successfully produced ceramsite by combining red mud, fly ash, and dredged sludge (DS), resulting in an impressive phosphate adsorption capacity of 9.84 mg/g. Lu et al. [4] also achieved the synthesis of porous ceramsite using dredged substrate and rice husk, specifically for removing Pb^2+^ from wastewater. Meanwhile, Li et al. [5] introduced a novel type of porous sintered ceramsite, utilizing municipal sludge, fly ash, and river sediments as raw materials, demonstrating its effectiveness in purifying water bodies from pollutants. Furthermore, Shao et al. [2] collaboratively developed ceramsite by incorporating sewage sludge and fly ash, focusing on the recovery of N and P from an actual wastewater treatment plant.

Electrolytic manganese residue (EMR) constitutes an acidic slag, a byproduct stemming from the electrolytic manufacturing process of manganese dioxide [6]. The production of EMR possesses substantial emissions, which necessitate vast land resources for stockpiling, and concurrently pose a considerable ecological hazard [7]. In light of these critical environmental concerns and the limited body of research on the development of materials derived from manganese slag for the purpose of heavy metal adsorption, it becomes imperative to devise a sustainable and economically viable solution. EMR, boasting an inherently high specific surface area [8], emerges as a promising candidate for conversion into a synthetic adsorbent material with minimal processing. Consequently, harnessing the latent potential of EMR represents a noteworthy avenue in the realm of environmental and materials science research.

The progression of industrialization and urbanization underscores the increasing significance and urgency of restoring and effectively managing urban lakes, as their condition profoundly impacts water quality and aquatic ecosystems [9]. In the engineered treatment process, the generation of dredged sludge (DS) solid waste is an inevitable byproduct [10,11]. This dredged sludge often contains a diverse array of contaminants, including inorganic compounds, heavy metals, and organic matter, which can pose environmental and human health threats [12,13]. Simultaneously, the rapid proliferation of water hyacinth (WH) raises significant concerns due to its potential to disrupt local ecosystems, causing issues such as watercourse clogs, water quality deterioration, and the endangerment of aquatic organisms [14]. On the other hand, WH is recognized as a substance that must be removed thanks to its adaptability to harsh environments and its remarkable capacity to adsorb pollutants from eutrophic waters [15]. The ongoing efforts to enhance water quality have resulted in the annual generation of substantial volumes of solid waste, including DS and WH. Various technologies have been developed to address single waste streams, encompassing methods such as incineration, anaerobic combustion, pyrolysis, and utilization as construction materials [16,17,18,19]. Among these approaches, the utilization of DS and WH as raw materials for ceramsite production has gained widespread recognition [20,21]. This is due to the fact that ceramsite production from dredged sludge or water hyacinth not only contributes to the efficient management of significant quantities of solid waste but also conserves natural clay resources. Moreover, it serves to immobilize the toxic substances inherent in the waste materials. Consequently, the synergistic utilization of dredged sludge, manganese sludge, and water hyacinth for ceramsite production emerges as a highly promising avenue. Nevertheless, it is noteworthy that the co-management of these three waste streams has received limited attention in the existing literature.

High-temperature sintered ceramsite and no-fire ceramsite methodologies present viable avenues for the synthesis of ceramsite using dredged sludge, manganese slag, and water hyacinth as feedstocks. It is essential to note that while no-fire ceramsite offers an environmentally friendly production route, it tends to exhibit limitations in terms of strength and durability, as elucidated in prior studies [22,23,24]. In contrast, sintered ceramsite emerges as a more robust alternative, displaying enhanced performance attributes. However, it is imperative to acknowledge that the high-temperature preparation of sintered ceramsite entails significant energy consumption and the release of substantial gaseous pollutants into the atmosphere. The no-fire ceramsite production process highlights a pressing need to not only reduce the energy requirements and pollutant emissions associated with the high-temperature sintering but also to elevate the overall performance characteristics of no-fire ceramsite materials. Addressing these imperatives is pivotal to advancing sustainable, efficient, and eco-friendly ceramsite production techniques for the utilization of EMR, DS, and WH, contributing to the broader realm of materials science and environmental engineering research. Therefore, a two-step ceramsite preparation process that integrates firing-free room-temperature curing and subsequent low-temperature firing was employed in this study, which can further improve the performance and service life of ceramsite with a low energy consumption.

The primary objective of this study is to employ EMR, DS, and WH as raw materials for the preparation of water treatment filter ceramsite with a two-step ceramsite preparation process to facilitate the efficient adsorption of phosphorus in wastewater. To evaluate the mechanical properties of the ceramsite, cylinder pressure strength is employed as a key evaluation parameter. Furthermore, the adsorption mechanism of ceramsite is elucidated by means of simulating both static adsorption and dynamic adsorption processes.

## 2. Materials and Methods

### 2.1. Materials

The electrolytic manganese residue used in this experiment came from an electroplating plant, the dredging sludge and water hyacinth were taken from the South Lake of Wuhan City, and the aluminate cement (AC) used as the binder was taken from Huaxin Cement Co, Wuhan, China. The raw materials were crushed, milled, and sieved to obtain powder with a particle size < 0.075 mm, and the chemical composition of each raw material was analyzed by X-ray fluorescence spectrometry (XRF); the data are shown in Table 1. The main chemical composition of the dredging mud is SiO_2_, which is the main source of the formation of ceramic granule skeleton. In addition, several metal oxides are present, including CaO, FeO, Mg_2_O_3_, Al_2_O_3_, Na_2_O, and K_2_O. Analytical grade chemicals such as potassium dihydrogen phosphate (KH_2_PO_4_), hydrochloric acid (HCl), and sodium hydroxide (NaOH) were purchased from local suppliers.

### 2.2. Preparation of Ceramsite

The EMR, DS, and WH were dried at 105 °C until they were completely free of moisture, followed by crushing and screening to collect 200-mesh passing powder for subsequent utilization. Granulation process of ceramsite was carried out in a rotary tilted disc of 400 mm diameter and 100 mm depth. The EMR, DS, AC, and WH were blended in accordance with a specific ratio, ensuring thorough and uniform mixing. Water, comprising approximately 45% to 55% of the overall mass of the dry materials, was gradually introduced to modulate the mixture’s viscosity until the resulting slurry attained a desired level of plasticity. The raw materials were then ground into round particles with diameters of 0.5–9 mm using a rotary granulator and immediately cured at a temperature of 20 °C with a relative humidity of 65% for several days to promote the hydration of AC. After curing, the ceramsite was oven-dried at 105 °C for 2 h to remove excessive moisture. The dried ceramsite was then placed into a box-type furnace for low-temperature burning at 500 °C with a heating rate of 4 °C/min for 1 h. The burning at the relatively low temperature foregoes the necessity for preheating. After cooling, the two-step ceramsite products were obtained.

### 2.3. Preparation of Phosphorus-Containing Wastewater

Accurately weigh 1.432 g KH_2_PO4, dissolve it with deionized water, transfer it to a 1000 mL volumetric flask to be fixed to the scale line, and shake well, resulting in a phosphate reserve solution (calculated as P); other concentrations of the solution can be directly diluted with the reserve solution.

### 2.4. Characterization

The cylinder compression strength was tested according to the standard of Light Aggregate and its Test Methods (GB/T17431.2-2010) [25]. In order to characterize the adsorption properties of the ceramsite, the specific surface area and pore volume were measured using a fully automatic specific surface area and porosity analyzer (ASAP2460, Micromeritics, Norcross, GA, USA). The chemical composition of the test raw materials was studied according to X-ray fluorescence spectrometry (XRF; Zetium, Panaco, Almelo, The Netherlands). Phosphate removal by DS-EMR during adsorption was analyzed using atomic absorption spectroscopy (AAS, CONTRAA-700, Jena, Germany). Micro-morphology and elemental composition were analyzed using scanning electron microscopy (SEM; TESCANMIRALMS, TESCAN, Brno, Czech Republic).

### 2.5. Static Adsorption

A total of 100 mL of phosphate solution was added to a conical flask and subsequently 0.1 g of ceramsite (over 200 mesh powder) was added to the conical flask and placed in a horizontal thermostatic oscillator for the experiments, with the vibration stroke adjusted to 26 mm and the rotational speed adjusted to 180 r/min. To study the adsorption kinetics and contact time of adsorption equilibrium, the adsorption reaction was carried out in this study at 25 °C for 100 mg/L of phosphorus. Then, adsorption isotherms were studied using phosphate solutions with initial concentrations of 50–1000 mg/L at the same temperature until the reaction reached equilibrium. In addition, the influencing factors such as the amount of ceramsite added (0.5, 1, 1.5, 2.0, 2.5, 3.0, 5.0 g/L) and pH (3–13) were also investigated. Calculations of adsorption rates, analysis of adsorption isotherms, and kinetic and thermodynamic calculations were obtained from previous literature [26,27]

The adsorption capacity ($q_{t}$, mg/g) of DS-EMR for phosphate adsorption was calculated according to Equation (1):

(1) $$q_{t}=\frac{\left(c_{0}-c_{t}\right)\cdot V}{m}$$

where *c*_0_ and *c_t_* denote the initial phosphate concentration and the phosphate concentration (mg/L) in the water samples taken at different time points, respectively, *V* is the volume of the solvent containing the water samples (L), and *m* is the mass of the DS-EMR (g).

The models widely used for adsorption kinetics are pseudo-first-order kinetic model and pseudo-second-order kinetic model, and the mathematical expressions are represented by Equations (2) and (3), respectively.

(2) $$q_{t}=q_{m}\left(1-e^{-k_{1}t}\right)$$

(3) $$q_{t}=\frac{{k_{2}q_{m}}^{2}t}{1+k_{2}q_{e}t}$$

where *q_t_* (mg/g) represents the adsorption capacity of phosphorus at time *t*, *q_m_* (mg/g) represents the adsorption capacity of DS-EMR at equilibrium, *k*_1_ is the pseudo-first-order absorption rate constant, and *k*_2_ is the pseudo-second-order kinetic rate constant.

The models widely used for adsorption isotherms are Langmuir and Freundlich models, and the mathematical expressions are represented by Equations (4) and (5), respectively.

(4) $$q_{e}=\frac{q_{m}C_{e}K_{L}}{1+C_{e}K}$$

(5) $$q_{e}=K_{F}C_{e}^{\frac{1}{n}}$$

where *q_e_* (mg/g) represents the adsorption capacity at DS-EMR adsorption equilibrium, *C_e_* (mg/L) denotes the phosphate concentration at adsorption equilibrium, *K_L_* is Langmuir constant, and *K_F_* is Freundlich constant.

### 2.6. Dynamic Adsorption

#### 2.6.1. Dynamic Test Setup

The dynamic adsorption experiments were performed using a homemade device. DS-EMR was used as the packing material, which was filled in the adsorption column with an inner diameter of 4 cm and a column height of 15 cm. The phosphate-containing water samples were diverted into the adsorption column by a peristaltic pump and flowed out of the column from top to bottom through the DS-EMR.

#### 2.6.2. Dynamic Tests

The initial concentration of phosphate was set to 100 mg/L, and was loaded into the influent storage bottle, the inlet rate of the peristaltic pump was adjusted to 5 mL/min, 15 mL/min, and 30 mL/min, respectively, and the penetration point of phosphate concentration of the effluent water was set to 30 mg/L. Adsorption saturation was recognized when the phosphate concentration of the effluent water was more than 90 mg/L. By measuring the effluent water under different flow conditions and plotting the penetration curves at different time intervals, accurate data of the phosphate concentration penetration curves were obtained. According to the penetration curve equation, the dynamic adsorption capacity *Q* (mg/g) in the absorption column can be measured, and the specific expression is shown in Equation (6).

(6) $$Q=\int_{0}^{V}\frac{\left(C_{0}-C_{1}\right)}{m}dV$$

where *Q* (mg/g) represents the dynamic adsorption capacity of the ceramsite in the adsorption column, *V* (mL) is the penetration volume at different flow rates, *C*_0_ (mg/L) is the nickel concentration at the time of influent, *C*_1_ (mg/L) is the nickel concentration at the time of effluent, and *m* (g) is the mass of ceramsite filled in the adsorption column.

## 3. Results and Discussion

### 3.1. Mechanical Behavior of Ceramsite

**Figure 1:**
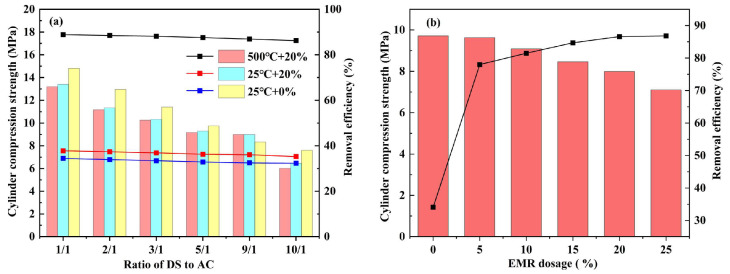
Effect of DS/AC ratio (**a**) and EMR dosage (**b**) on ceramsite cylinder compression strength and P^+5^ removal efficiency.

Figure 1 illustrates the influence of different ratios of the dredging substrate, aluminate cement addition, and EMR on the compression strength and P^+5^ removal efficiency of ceramsite. It can be observed from Figure 1a that as the sludge/cement ratio shifts from 3/1 to 5/1 and then to 9/1, the change in compressive strength of the ceramsite remains minimal, essentially maintaining a consistent level. However, when the sludge/cement ratio is increased to 10/1, a significant drop in the compressive strength of the ceramsite becomes evident. This decrease may be attributed to the excessive sludge content in the pellet composition, resulting in an insufficient proportion of aluminate cement and subsequently leading to instability in pellet strength [28]. During firing at 500 °C, the combustion of biomass generates gases that create additional pores both internally and on the surface of the ceramsite, thereby greatly enhancing the phosphorus adsorption capacity of the ceramsite [29,30]. Considering the impact of various sludge/cement ratios on the compressive strength and phosphorus adsorption performance of the ceramsite, a sludge/cement ratio of 9/1 was chosen for subsequent experiments. Furthermore, by substituting a portion of the dredged substrate with EMR, it was observed from Figure 1b that the removal efficiency of composite ceramic granules for phosphorus initially increased and then decreased with higher levels of manganese slag doping. The optimal doping level for manganese slag was determined to be 20%. Consequently, the optimal ratio of composite pellets in this study was established as dredging mud: electrolytic manganese slag: aluminate cement = 7:2:1, and an additional 5 wt% of WH was incorporated as a pore-forming additive (referred to as DS-EMR in the following experiments).

### 3.2. Effect of pH and Ceramsite Dosage on Adsorption

**Figure 2:**
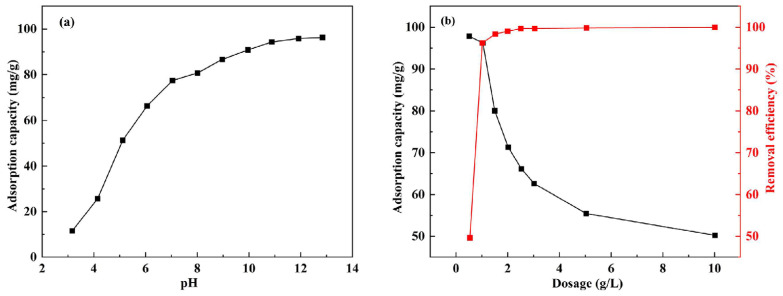
Fitting plots of influence of the (**a**) pH, (**b**) dosage on adsorption.

In adsorption processes, pH plays a critical role in influencing the charges and functional group states of the adsorbent [31,32]. In Figure 2a, the graph illustrates the adsorption capacity of ceramsite in relation to various initial pH values of P^+5^ solutions at a dosage of 0.5 g/L. As depicted in Figure 2a, the adsorption capacity exhibits an upward trend with increasing pH. This can be primarily attributed to the protonation of the material’s surface at lower pH levels, resulting in a positive charge, which leads to electrostatic repulsion with metal cations [33,34]. Conversely, as pH increases, the adsorbent surface undergoes ionization of -OH groups, causing the adsorption of P^+5^ ions onto the DS-EMR surface through electrostatic interactions until the surface is neutralized [35,36]. Therefore, the optimal value of the pH is 5.

The adsorbent dosage is a crucial factor in the adsorption process. In Figure 2b, the adsorption capacity of DS-EMR is depicted in response to varying ceramsite dosages at pH = 5. As the dosage of DS-EMR increased, the removal efficiency gradually rose, eventually stabilizing. Notably, the adsorption efficiency exhibited rapid growth when the dosage increased from 0.5 to 1 g/L due to an increase in the number of available adsorption sites [37]. However, when the dosage exceeded 1 g/L, the removal rate of P^+5^ increased at a slower rate. It reached a removal efficiency of 99% at a dosage of 2 g/L. It is worth noting that the adsorption capacity decreases as the adsorbent dosage increases, which is primarily attributed to the rise in active sites resulting in a reduction in the adsorption specific surface area, and creates challenges in reaching saturation. In summary, the optimal adsorption dosage of 1 g/L can be determined, which exhibited an adsorption capacity of up to 94.19 mg/g, and the removal rate of P^+5^ also reached 96.26%.

### 3.3. Characterization of DS-EMR Ceramsite

#### 3.3.1. Pore Characteristics

**Figure 3:**
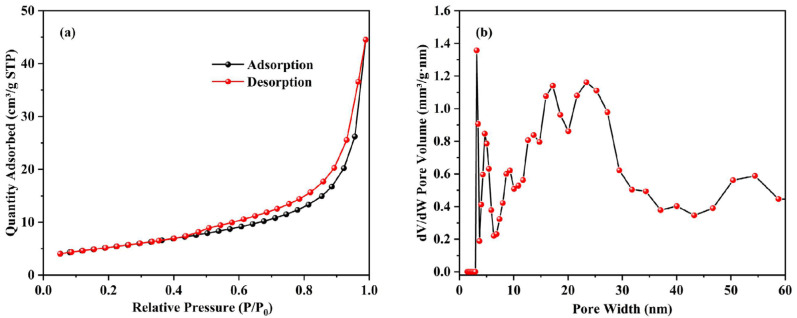
Pore characteristics of ceramsite through (**a**) nitrogen adsorption–desorption isotherm and (**b**) pore size distribution.

Figure 3a presents the results of the nitrogen adsorption–desorption test for DS-EMR ceramsite. At P/P_0_ values ranging from 0.5 to 1.0, a noticeable hysteresis loop was observed in the absorption and desorption curves. This suggests a significant increase in nitrogen adsorption by the ceramsite within this range, demonstrating that the DS-EMR ceramsite possesses a broad pore size distribution [38]. Upon comparing the types of hysteresis loops, it becomes clear from Figure 3a that an H3-type hysteresis loop is present. This type is typically observed in clay materials and is indicative of the presence of lamellar or fissure pores [39]. Figure 3b displays the pore size distribution curve for the ceramsite. Analysis reveals that the pores in DS-EMR ceramsite primarily range from 3 to 60 nm, with a predominant concentration in the 3–10 nm range. This suggests that DS-EMR ceramsite is a mesoporous material characterized by an uneven distribution of pore sizes, with a tendency toward smaller pores.

#### 3.3.2. Micromorphology Analysis

**Figure 4:**
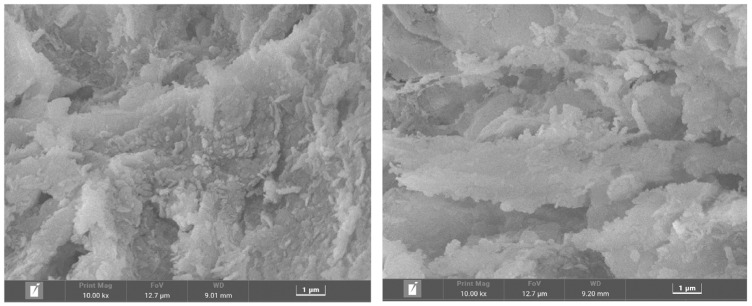
SEM image of DS-EMR ceramsite.

Figure 4 shows the microstructure of DS-EMR. It can be seen that calcination at 500 °C produces floccules and pores due to the devolatilization of WH. This type of surface of the ceramsite, which contained adequate pores and cracks, facilitated the adsorption of P^+5^.

### 3.4. Adsorption Kinetics

**Figure 5:**
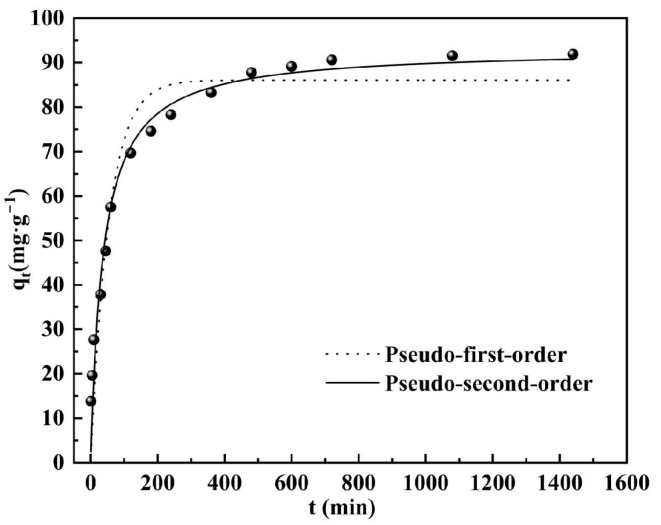
Fitting results of adsorption kinetics.

The process of phosphate adsorption by DS-EMR was analyzed using both the pseudo-first-order and pseudo-second-order kinetic models, as depicted in Figure 5. Upon fitting the data, the kinetic constants, *k*_1_ for the pseudo-first-order model and *k*_2_ for the pseudo-second-order model, were determined and are presented in Table 2.

The correlation coefficients (R^2^) for the pseudo-first-order and pseudo-second-order kinetic models were found to be 0.8595 and 0.9968, respectively, highlighting the particularly strong correlation coefficients associated with the pseudo-second-order model. Furthermore, the maximum adsorption capacity (*q_m_)* calculated using the pseudo-second-order model closely aligned with the experimental values. These findings suggest that the pseudo-second-order kinetic model is better suited to describing the adsorption behavior of DS-EMR on P^+5^, with the removal process being primarily attributed to chemisorption [40].

### 3.5. Adsorption Isotherm

**Figure 6:**
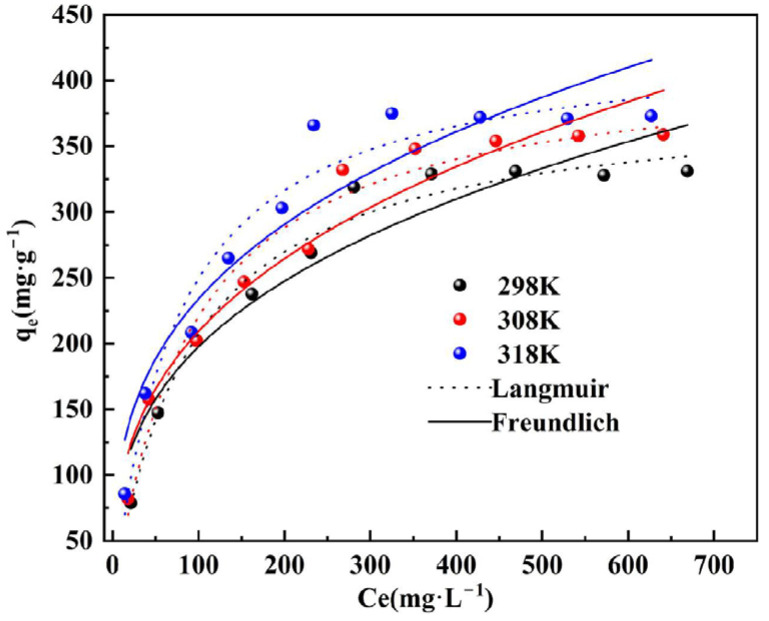
Fitting diagram of adsorption isotherm.

Upon analyzing the experimental data in Figure 6 and applying both the Langmuir and Freundlich models, it was evident that both models effectively characterized the isothermal adsorption behavior of phosphorus by DS-EMR ceramic granules. The fitting results for both models are detailed in Table 3.

Notably, at various temperatures, the R^2^ values for the Langmuir model exceeded 0.95, consistently surpassing the R^2^ values of the Freundlich model. This trend indicates that the Langmuir model offers a more precise description of the isothermal adsorption behavior of phosphorus by DS-EMR ceramsite [41,42]. Furthermore, the parameter 1/n in the Freundlich isothermal adsorption model exhibited values of 0.325, 0.339, and 0.313 at 25 °C, 35 °C, and 45 °C, respectively. These values, falling within the range of 0 to 1, signify that DS-EMR ceramsite possesses a high and efficient capacity for phosphorus adsorption.

### 3.6. Dynamic Adsorption

#### 3.6.1. Penetration Curves

**Figure 7:**
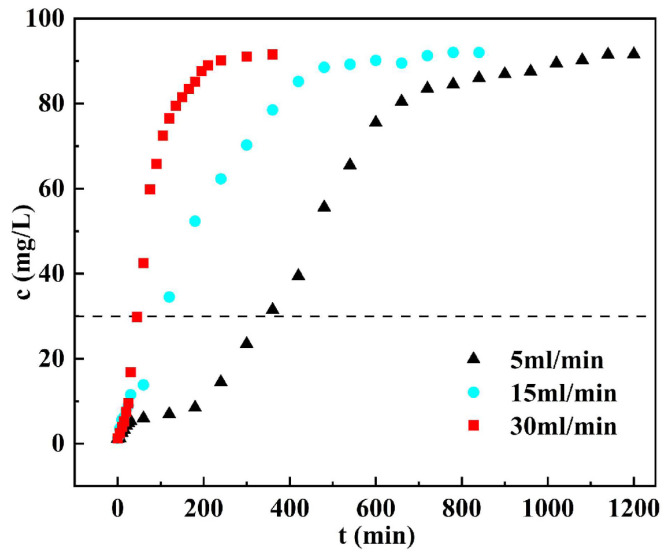
Adsorption column penetration curve during dynamic adsorption of ceramsite.

The penetration curve model refers to the relationship between the concentration of solutes in the liquid flowing through an adsorption column and time over a certain period. By analyzing the breakthrough curve, one can understand the transfer and adsorption process of solutes in the adsorption column and also assess the performance and effectiveness of the column [43,44]. This curve facilitates the analysis of the pore size distribution, pore structure, and kinetic characteristics of the adsorption process. To generate accurate phosphorus concentration penetration curves, measurements of effluent under various flow conditions and at distinct time intervals were conducted, as illustrated in Figure 7.

The volume of 125.66 mL can be calculated based on the inner diameter and width of the absorption column. Additionally, hydraulic residence times at different flow rates (5 mL/min, 15 mL/min, and 30 mL/min) were determined to be 25.13 min, 8.38 min, and 4.19 min, respectively. Figure 7 provides valuable insights into the impact of various flow rates on the dynamic adsorption of phosphorus by DS-EMR ceramsite. Notably, different flow rates significantly influence the phosphorus adsorption process. For instance, at a flow rate of 5 mL/min, the point of phosphorus adsorption saturation by DS-EMR ceramsite was reached after 360 min, with approximately 1800 mL of phosphorus passing through the ceramic adsorption column. In contrast, at a flow rate of 15 mL/min, saturation occurred after 110 min, with a phosphorus penetration volume of about 1650 mL. When the flow rate was set to 30 mL/min, saturation was achieved in just 45 min, and the phosphorus penetration volume was approximately 1350 mL. It is evident that higher flow rates result in shorter residence times in the adsorption column, limiting the contact between the phosphorus solution and DS-EMR ceramsite, thereby reducing phosphorus removal efficiency [45]. Consequently, excessively high water flow rates can lead to reduced DS-EMR ceramsite utilization.

#### 3.6.2. Dynamic Adsorption Models

To elucidate the adsorption mechanism of DS-EMR-adsorbed phosphorus, the performance and parameters of the adsorption columns were predicted using the Adams–Bohart model, the Thomas model, the Yan model, and the Yoon–Nelson model. Their mathematical forms are shown in Equations (7) to (10), respectively [46,47].

(7) $$\ln\frac{C_{t}}{C_{0}}=k_{1}C_{0}t-k_{1}N_{0}\frac{H}{V}$$

(8) $$\ln\left(\frac{C_{0}}{C_{t}}-1\right)=k_{W}q_{W}\frac{w}{Q}-k_{W}C_{0}t$$

(9) $$ln(\frac{C_{0}}{C_{t}}-1)=\frac{k_{4}C_{0}}{Q}ln\frac{k_{4}Q_{Y}W}{Q}-\frac{k_{4}C_{0}}{Q}lnQ_{t}$$

(10) $$\ln\frac{C_{t}}{C_{0}-C_{t}}=k_{5}t-t_{\frac{1}{2}}k_{5}$$

where *k*_1_ is the mass transfer coefficient; *N*_0_ is the dynamic adsorption capacity, mg/L; *H* is the adsorption column pellet height, cm; *V* is the flow rate, mL/min; *K_W_* is Thomas’ constant; *q_W_* (mg/g) is the adsorption capacity of phosphorus; *W* (g) is the amount of adsorbent in the column; *Q* (mL/min) is the flow rate; *k*_4_ is the reaction rate constant; *Q_Y_* (mg/g) is the maximum adsorption capacity; *Q_t_* (mg/g) is the adsorption capacity at time *t*; *k*_5_ is the reaction rate constant; and *t*_1/2_ (min) is the time for the concentration to decrease to 50%.

**Figure 8:**
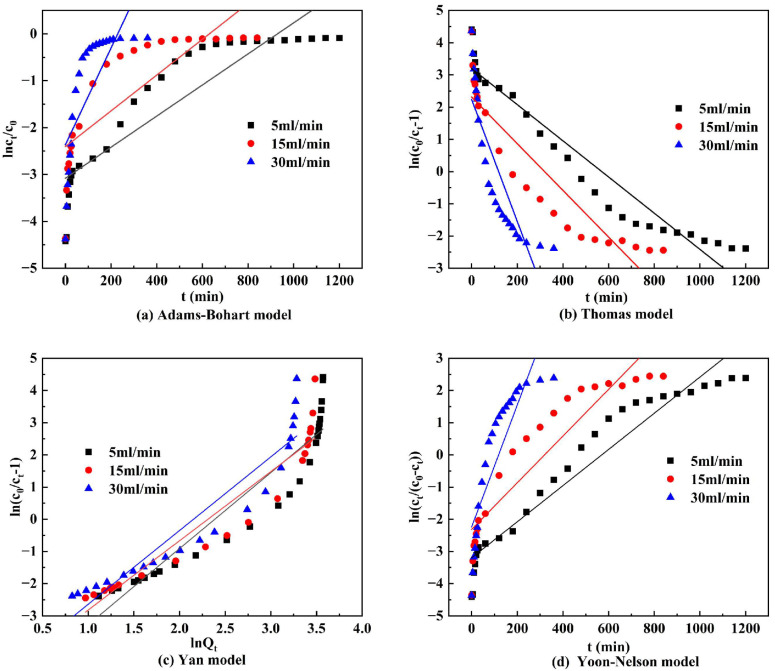
Fitting results of dynamic adsorption model.

As shown in Figure 8, based on the fitted curves, it is evident that the DS-EMR ceramsite aligns more closely with the fitted curves of the Thomas, Yan, and Yoon–Nelson models. This alignment suggests that the ceramsite can effectively meet the assumptions of the Langmuir adsorption isotherm and pseudo-second-order reaction kinetics during dynamic adsorption for the removal of phosphorus [48].

## 4. Conclusions

In this study, a composite dredged substrate–electrolytic manganese residue (DS-EMR) was synthesized using dredged substrate, aluminate cement, water hyacinth, and manganese slag with a high specific surface area, which enhances the removal of phosphorus from the aqueous medium through the concept of “waste for waste”.

1. Through the mechanical and adsorption properties tests, the optimal ceramsite (DS-EMR) was prepared by mixing EMR, DS, and AC at a ratio of 7:2:1 by mass, adding 5 wt% WH as a pore-forming additive, and then sintering at 500 °C.
2. Batch adsorption experiments of DS-EMR demonstrated that, at a dosage of 1 g/L, pH = 4 exhibited an adsorption capacity of up to 94.19 mg/g with 96.26% phosphorus removal, indicating that DS-EMR has a significant effect and application prospect for phosphorus removal from wastewater.
3. The static adsorption tests showed that the pseudo-second-order kinetic model and Langmuir adsorption isotherm model gave the best fit, indicating that the monolayer chemisorption interactions may play a dominant role in the adsorption of phosphorus by DS-EMR.
4. The dynamic adsorption test used four simplified dynamic adsorption models to predict the experimental results, and the DS-EMR ceramic grains fitted well with the Thomas, Yan, and Yoon–Nelson models, indicating that DS-EMR ceramic grains were also able to satisfy the assumptions of the Langmuir adsorption isotherm and pseudo-second-order adsorption kinetics during the dynamic adsorption of phosphorus removal.

## Figures and Tables

**Table 1:** Chemical composition analysis of raw materials (%).

	DS	EMR	WH	AC
SiO_2_	62.19	23.32	2.67	0.57
CaO	3.04	11.36	1.50	31.33
Fe_2_O_3_	4.86	2.84	0.29	0.12
MgO	1.86	2.77	0.68	0.34
Al_2_O_3_	14.47	7.88	0.97	66.93
Cl	/	/	2.35	/
SO_3_	0.08	26.73	0.79	0.01
Na_2_O	1.10	0.39	0.18	0.35
K_2_O	2.52	1.64	0.47	0.02
MnO	/	5.42	/	/

**Table 2:** The parameter of the pseudo-first-order and the pseudo-second-order.

Pseudo-First-Order			Pseudo-Second-Order		
*q_e_*/mg·g^−1^	*k*_1_	R^2^	*q_e_*/mg·g^−1^	*k*_2_	R^2^
7859.51	0.00289	0.8595	94.0734	0.00031	0.9968

**Table 3:** Fitting parameters of Langmuir and Freundlich model.

T/°C	Langmuir Model			Freundlich Model		
	*q_m_*	*K_L_*	R^2^	*K_F_*	n	R^2^
25	386.65	0.01154	0.9787	44.28	3.08	0.9034
35	415.01	0.01136	0.9650	43.96	2.95	0.9350
45	432.25	0.01365	0.9545	55.29	3.19	0.8880

## Data Availability

Data are contained within the article.
